# Dysregulated fibroblast–immune crosstalk drives statin-associated erectile dysfunction: integrative evidence from pharmacovigilance and single-cell transcriptomics

**DOI:** 10.3389/fphar.2026.1787039

**Published:** 2026-03-24

**Authors:** Yang Zhang, Song Wen, Yanping Zhu, Qinyong Zhang, Pengyi Zheng, Xiaohui Wang, Shuangwu Lv

**Affiliations:** 1 Department of Urology Surgery, The First Affiliated Hospital, and College of Clinical Medicine of Henan University of Science and Technology, Luoyang, China; 2 Department of Oncology, Ganzhou Cancer Hospital, The Affiliated Cancer Hospital of Gannan Medical University, Ganzhou, Jiangxi, China

**Keywords:** erectile dysfunction, Fibroblast–immune interactions, inflammatory microenvironment, pharmacovigilance, statins

## Abstract

**Background:**

Mounting evidence points to a potential link between statin therapy and erectile dysfunction (ED). However, the underlying mechanisms remain elusive, particularly concerning potential statin disruption of the inflammatory and immunofibrotic microenvironment within erectile tissue. This study sought to elucidate statin-associated ED and identify key molecular and cellular mediators driving this process.

**Methods:**

An integrative strategy merging real-world pharmacovigilance, network pharmacology, and toxicological analyses was deployed to explore drug-associated ED. Drug-related and disease-associated targets were intersected and scrutinized using protein–protein interaction (PPI) networks to pinpoint key regulatory genes. Gene expression patterns were evaluated through bioinformatics analyses and validated by reverse transcription–quantitative polymerase chain reaction (RT-qPCR). Molecular docking and molecular dynamics (MD) simulations assessed drug–target interaction stability. Furthermore, single-cell RNA sequencing (scRNA-seq) analysis characterized cell-type-specific alterations and intercellular communication dynamics within erectile tissue.

**Results:**

Disproportionality analysis identified atorvastatin (ROR = 3.36, 95% CI = 3.04–3.70) and rosuvastatin (ROR = 3.22, 95% CI = 2.81–3.69) as statins significantly linked to ED, exhibiting moderate predicted toxicity. Four key genes—FGFR1, SERPINE1, TGFB2, and TGFBR2—emerged as potential mediators connecting statin exposure to ED. FGFR1 expression plunged significantly, while SERPINE1 expression surged markedly in ED samples, findings consistently observed in both transcriptomic analyses and RT-qPCR validation. Molecular docking and MD simulations demonstrated stable binding between atorvastatin and FGFR1. Notably, scRNA-seq analysis revealed fibroblasts as central immunomodulatory cells in ED, with their intercellular communication dysregulation closely related to the abnormal activation of the FGFR1 axis, which further mediates the local immunofibrotic disorder of the corpus cavernosum.

**Conclusion:**

This study delivers convergent evidence that atorvastatin and rosuvastatin associate with ED and implicates dysregulated inflammatory and immunofibrotic remodeling of erectile tissue as a potential underlying mechanism. The identified key genes and fibroblast-centered cellular interactions yield fresh insights into statin-associated ED and highlight promising molecular targets for future translational and immunomodulatory therapeutic strategies.

## 1 Introduction

Erectile dysfunction (ED) is a prevalent disorder among men of all ages, characterized by the chronic inability to attain or sustain an erection enough for acceptable sexual intercourse (Wang et al., 2023). Approximately 15% of men are predicted to encounter this illness annually (Chung et al., 2023). The erectile reflex is meticulously governed by a sophisticated central-peripheral network that includes the cerebral cortex, spinal cord, peripheral nerves, and tissues, with the hypothalamus and hippocampus being pivotal in the integration of sexual function and erection (Sheibani et al., 2022). The etiology of ED is extensive, conventionally classified into five primary categories: psychogenic, neurological, vascular, hormonal, and drug-associated (Zang et al., 2022). Pharmacological ED, although curable, is frequently disregarded and represents a therapeutic “invisible” difficulty. Studies indicate that primary hypertension drugs, including thiazides and beta-blockers, may adversely affect sexual function (Corona et al., 2025). Furthermore, a survey indicated that 28% of patients with ED ascribed the problem to drug usage (Tian et al., 2025). Consequently, creating a swift identification checklist for “drug-associated ED” is essential for enhancing drug management strategies in clinical practice.

Among drug-induced ED, statins—despite their cardiovascular benefits—have emerged as potential contributors, though their mechanisms remain poorly understood. For decades, statins have been considered essential drugs for cholesterol lowering and cardiovascular prevention (Joentausta et al., 2023). As inhibitors of 3-hydroxy-3-methylglutaryl-coenzyme A reductase, they markedly diminish cholesterol and triglyceride levels by obstructing cholesterol production in the liver (Chuma et al., 2022). Nevertheless, previous research suggests that these drugs may worsen ED (Omolaoye et al., 2022). Atorvastatin, a commonly utilized lipid-lowering statin, concurrently inhibits the proliferation and migration of vascular smooth muscle cells while promoting apoptosis (Beltrán Romero et al., 2021). Joentausta et al. indicated a marginally elevated likelihood of commencing ED treatment among atorvastatin users post-radical prostatectomy (Joentausta et al., 2023). Likewise, rosuvastatin exhibits direct beneficial effects on lipid profiles, in addition to possessing anti-inflammatory, antioxidant, anti-thrombotic, and vasoprotective attributes (Mostaza and Escobar, 2024). Nonetheless, the administration of rosuvastatin has been linked to many side effects, such as back pain and diminished appetite (Li Q. et al., 2025). This study seeks to identify the potential target mechanisms and functional roles of atorvastatin and rosuvastatin in relation to ED.

FDA Adverse Event Reporting System (FAERS) database encompasses millions of case reports concerning adverse drug events (ADEs), making it one of the largest pharmacovigilance databases globally (Yin et al., 2022; Horinouchi et al., 2018). This study conducted a systematic pharmacovigilance analysis using FAERS database to identify high-risk drugs linked to ED. By integrating network pharmacology and toxicology techniques, we swiftly clarified the hazardous processes of these drugs. Additionally, key genes were identified by drug-disease interactions, whose expression was validated by reverse transcription-quantitative polymerase chain reaction (RT-qPCR). Ultimately, single-cell RNA sequencing (scRNA-seq) research was performed to reveal single-cell features ED. These analyses aimed to thoroughly characterize the risk profiles of ED-associated drugs, describe their toxicological features related to ED, and establish a foundation for precision therapies and target discovery in ED.

## 2 Materials and methods

### 2.1 Data source and processing

**FIGURE 1:**
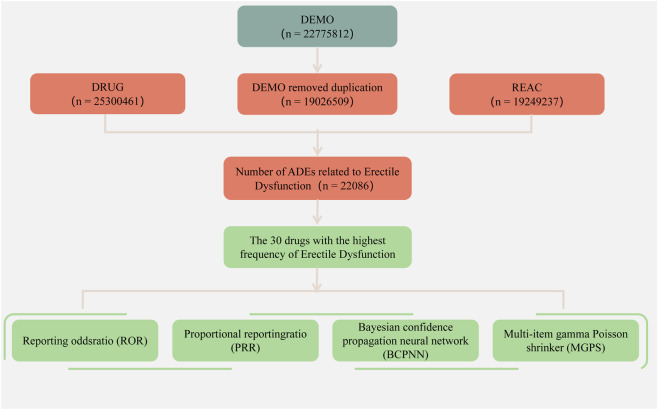
The flow-process diagram of erectile dysfunction (ED) from FAERS database.

This study aimed to analyze the drugs associated with ED to identify those with positive signals that contribute to ED. ADEs data used in this study were downloaded from the FAERS database (https://www.fda.gov/drugs) and covered the period from Q1 2004 to Q1 2025. These data contained DEMO (patient demographics and management information), REAC (ADE information), DRUG (drug information), OUTC (patient outcomes), RPSR (reporting source), THER (treatment initiation and cessation dates for reported drugs), and INDI (indications for drug administration) (Zhang L. et al., 2023). In accordance with FDA guidelines, duplicates were removed using the following criteria: (1) the most recent FDA-DT was selected when CASEIDs matched, (2) the higher PRIMARYID was chosen when both CASEIDs and FDA_DTs were identical (Wang et al., 2024). To standardize drug’s names, RxNorm was applied. Furthermore, ADEs were standardized by searching system organ class (SOC) and preferred terms (PT) of Standardized MedDRA Queries (SMQs) from the Medical Dictionary for Regulatory Activities (MedDRA). The entire study design flowchart was shown in Figure 1.

ED-related transcriptome (GSE10804) and scRNA-seq datasets (GSE206528) were downloaded from the Gene Expression Omnibus (GEO, https://www.ncbi.nlm.nih.gov/gds/) database. GSE10804 dataset, including 7 control and 5 ED human cavernosal endothelial cell samples, was sequenced by GPL571. GSE206528 dataset (platform: GPL24676) comprised of 3 control and 5 ED human corpus cavernosum samples. To avoid systemic biases caused by cellular composition differences between heterogeneous datasets, strict bias control strategies were adopted in this study: first, the R package sva was used to eliminate technical batch effects from different sequencing platforms and sample processing protocols; second, a cell type-stratified analysis strategy was strictly implemented—we did not conduct direct global comparison between bulk endothelial cell data and whole-tissue scRNA-seq data, but extracted the endothelial cell subpopulation from GSE206528 for independent expression analysis after accurate cell type annotation. The downregulation of FGFR1 in endothelial cells was interpreted in a cell type-specific manner across datasets, and its expression trend in the endothelial cell subpopulation of scRNA-seq data was consistent with that in the bulk transcriptomic data of purified cavernosal endothelial cells, which ensured the reliability of cross-dataset results.

### 2.2 Disproportionality analysis

To assess the associations between ED and drugs, disproportionality analysis was performed using multiple methods, including reporting odds ratio (ROR), proportional reporting ratio (PRR), Bayesian confidence propagation neural network (BCPNN), and multi-item gamma Poisson shrinker (MGPS) (Jiang et al., 2024). These algorithms were based on a 2 × 2 contingency table (Supplementary Table S1), with formulas outlined in Supplementary Table S2. Positive signals were considered valid if the number of reports was at least 3, the lower limit of the ROR 95% confidence interval (CI) should be greater than 1, PRR ≥2, χ^2^ ≥ 4, IC_025_ > 0, and EBGM_05_ > 2. A larger signal value (i.e., ROR) indicates a stronger association between the ED and the suspected drugs, though it does not imply a biologically causal relationship. By this way, drugs-associated ED were selected.

### 2.3 Prediction of drug targets and ED targets

The pharmmapper (https://www.lilab-ecust.cn/pharmmapper/), swisstarget (http://swisstargetprediction.ch/), and targetnet databases (http://targetnet.scbdd.com/home/index/) were utilized to forecast targets for drugs obtained above. Furthermore, OMIM (http://www.omim.org/), STRING (http://string-db.org/), and GENECARDS databases (https://www.genecards.org/) were utilized to predict ED-related targets using the key word “ERECTILE DYSFUNCTION”. After merging and removing duplication, drug- and ED-targets were acquired for further analysis.

### 2.4 Differential expression analysis

Differentially expressed genes1 (DEGs1) between ED and control samples were discovered utilizing “limma” package (version 3.54.2) (Ritchie et al., 2015) with the criteria of |log_2_fold change (FC)| > 0.5 and *P* < 0.05. The volcano plot of DEGs1 was produced by “ggplot2” package (version 3.5.1) (Cao et al., 2023), whereas the heat map for top 20 up- and downregulated DEGs1 was constructed using “pheatmap” package (version 1.0.12) (Zhang X. et al., 2023). In this study, all P < 0.05 thresholds for DEGs screening signify the false discovery rate (FDR)-corrected statistical significance, with multiple testing correction employed to minimize false positives.

### 2.5 Identification and analysis for intersection genes correlated with drugs and ED

The overlap of drug-targets, ED-targets, and DEGs1 was determined to yield intersection genes. To investigate the functional role of intersection genes, GO and KEGG enrichment analyses were conducted using “clusterProfiler” package (version 4.6.2) (Yu et al., 2012). Additionally, the interaction of intersection genes at protein level was explored, and a protein-protein interaction (PPI) network was constructed using STRING database. Finally, a drug-intersection gene-ED network was created by Cytoscape software (version 3.10.2) (Shannon et al., 2003).

### 2.6 Identification and analysis of key genes

Based on the PPI network of intersection genes, 4 algorithms-Maximal Clique Centrality (MCC), Maximum Neighborhood Component (MNC), Density of Maximum Neighborhood Component (DMNC), and Degree-were employ to ascertain top 10 genes for each algorithm. The convergence of top 10 genes for 4 algorithms was regarded as key genes. The expression of these key genes was evaluated in the GSE10804 dataset. Furthermore, the drug-key gene-KEGG pathway network was also built using Cytoscape software.

### 2.7 Cell culture and treatment

Immortalized rat cavernous endothelial cells, which were cultured in specialized culture medium, were purchased from Immocell Biotechnology. These cells were maintained in the atmosphere with 5% CO_2_ at 37 °C. Furthermore, these cells were treated with 0.3 mM palmitic acid (MCE, HY-N0830) for 24 h to establish ED cell model. Palmitic acid was chosen to establish an *in vitro* endothelial injury model relevant to ED, as it can induce lipotoxic damage in corpus cavernosum endothelial cells, which is related to endothelial dysfunction involved in ED pathogenesis. This *in vitro* model has been employed to study endothelial injury–associated ED, and is suitable for evaluating the functional roles of key genes in the context of endothelial dysfunction relevant to ED.

### 2.8 Reverse transcription-quantitative polymerase chain reaction (RT-qPCR)

Total RNA was extracted from cells using FastPure Complex Tissue/Cell Total RNA Isolation Kit (Vazyme, RC113-01). Its concentration was measured with a Nanodrop-500, and reverse transcription to cDNA was performed using ABScript III RT Master Mix for RT-qPCR with gDNA Remover (Abclonal, RK20429). RT-qPCR was conducted with Genious 2X SYBR Green Fast RT-qPCR Mix kit (ABclonal, Wuhan) following manufacturer’s instructions. Biological and technical triplicates were used to ensure result reliability. The mRNA levels were normalized to GAPDH, and relative gene quantification was performed using 2^−ΔΔCT^ approach. Primer sequences were listed in Table 1.

**TABLE 1:** The sequence of primers.

Primers	Sequence 5′-3′
GAPDH-f	TGG​TGA​AGG​TCG​GTG​TGA​AC
GAPDH-r	TTC​TCA​GCC​TTG​ACT​GTG​CC
Serpine1-F	CTT​CTT​AGA​GGC​CAG​CAC​CC
Serpine1-R	GAT​GTC​GTA​CTC​GTG​CCC​AT
Tgfb2-F	TCC​CCT​CCG​AAA​ATG​CCA​TC
Tgfb2-R	GAG​ACA​TCG​AAG​CGG​ACG​AT
Tgfbr2-F	CCC​AAG​TCG​GTT​AAC​AGC​GA
Tgfbr2-R	TGT​CGT​TCT​TCC​CCA​CAC​G
Fgfr1-F	AGG​GCA​ACT​ACA​CCT​GCA​TC
Fgfr1-R	TAC​GGC​AAG​TTG​TCT​GGA​CC

### 2.9 Molecular docking and molecular dynamics (MD) analyses

To explore the associations between key genes with corresponding drugs, molecular docking analysis was carried out. The 3D structure (SDF file) of drugs was downloaded from PubChem database, and converted to PDB format using Babel GUI. The 3D structure for key gene protein was obtained from uniport database. AutoDock software was used to remove water molecules and small molecular ligands, along with molecular docking analysis. PyMol was then employed to visualize the docking results.

Based on the results of molecular docking analysis, we selected drug-key gene interaction pairs with the lowest binding energy to perform MD analysis to understand their binding modes. MD simulations were performed using the Gromacs2022 software, with the GAFF force field for small molecule, while AMBER14SB force field and the TIP3P water model for proteins. All hydrogen-containing bonds were frozen via the LINCS algorithm, allowing a 2-fs integration step. Long-range electrostatics were treated with Particle-mesh Ewald (PME) (cutoff 1.3 nm), whereas the cutoff value of non-hydrogen interactions was 10Å and refreshed every 10 steps. The system was thermostated at 298 K with V-rescale and barostated at 1 bar with Berendsen coupling. After 100 ps NVT and NPT equilibration, a 100-ns production run was carried out, saving snapshots every 10 ps. Trajectory analysis was conducted in VMD and PyMOL, and protein-ligand binding free energies were estimated with g_mmpbsa. The system parameters, such as root mean square deviation (RMSD), root mean square fluctuation (RMSF), radius of gyration (Rg), Hbond, and solvent accessible surface area (SASA), were determined.

### 2.10 Toxicity analysis and ADMET evaluation

The SwissADME database (http://www.swissadme.ch/) was used to analyze the absorption, distribution, metabolism, excretion, and toxicity (ADMET) properties of obtained drugs. ProTox-III database (https://tox.charite.de/protox3/) was then consulted to predict the toxicity of drugs.

### 2.11 Gene set enrichment analysis (GSEA)

To elucidate the function of key genes in ED, GSEA was conducted using “clusterProfiler” package with the background gene set “c2. cp.kegg.v2024.1. Hs.symbols.gmt”, acquired from the GSEA website (http://www.gsea-MSigdb.org/gsea/msigdb). Spearman analysis was used to calculate correlation coefficient between key genes and all genes in GSE10804 dataset utilizing “psych” package (version 2.4.3) (Revelle, 2024) and ranked. The thresholds for GSEA were established at |NES| > 1 and *P* < 0.05.

### 2.12 Regulatory relationships prediction for key genes

The miRDB and starbase databases were utilized to predict miRNAs correlated with key genes, and lncRNAs associated with miRNAs, respectively. The screening threshold for lncRNAs was clipExpNum >20. Regulatory network for key gene-miRNA-lncRNA was created via Cytoscape.

### 2.13 ScRNA-seq analysis

Single cell signatures of ED samples were assessed by scRNA-seq analysis utilizing the “Seurat” package (version 5.1.0) (Hao et al., 2024). Quality control was implemented according to the following criteria: (1) genes covered by fewer than 3 cells were eliminated; (2) cells exhibiting greater than 10% mitochondrial gene were omitted; (3) cells with genes ranging from 200 to 5,000, alongside count between 200 and 20,000 were preserved. The vst method in FindVariableFeatures function was carried out to extract top 2,000 highly variable genes. ScaleData function was used to scale scRNA-seq data, and statistically significant principle components (PCs) were determined by JackStrawPlot function. Top 30 PCs were subsequently chosen for further analysis. The FindNeighbors and FindClusters functions in “Seurat” package were employed to identify small clusters with resolution = 0.2, which were visualized by uniform manifold approximation and projection (UMAP) method. According to the marker genes for each cell cluster (Zhao et al., 2022), cell types were then annotated. To reduce the influence of doublets for the analysis, “DoubletFinder” package (version 2.0.4) (Zhu et al., 2024) was used to remove the doublets in GSE206528 dataset. The expression of key genes in cell types was then compared between ED and control samples using Wilcoxon test (*P* < 0.05).

To identify cell types correlated with ED, single cell data were integrated with bulk transcriptome data using “Scissor” package. The parameter for Scissor function was alpha = 0.01 and family = “binomial”, and Scissor+ and Scissor-cells were identified. Alpha = 0.01 was rigorously set as the statistical significance level to minimize the false positive rate in cell-type association analysis; the binomial family was selected because the outcome variable (ED versus normal control) represents a binary categorical state, making it ideally suited for the binomial distribution model. Scissor+ was positively correlated with ED, while Scissor-was negatively associated with ED. Scissor+ and Scissor-cells were extracted, and differential expression analysis was conducted across these two groups using “FindMarkers” function to identify DEGs2 with |log_2_FC| ≥ 1, percentage ≥0.1, and adj. *P* < 0.05. To assess the functions of DEGs2, GO and KEGG enrichment analyses were conducted with adj. *P* < 0.05. All P < 0.05 referring to FDR-corrected values.

### 2.14 Pseudotime analysis and cell communication

The key cell type was selected based on Scissor analysis and the expression levels of core genes-those identified by the lowest binding energy in molecular docking. Key cell type was further divided into high and low core gene expression groups relied on the expression of core gene. Differential expression analysis was conducted across these expression groups, |log_2_FC| was the ordering criteria. GSEA was then conducted to enrich pathways with |NES| > 1 and *P* < 0.05. Furthermore, pseudotime analysis was performed on the key cell type using “Monocle” package (version 2.26.0) (Qiu et al., 2017) to explore the differentiation of cells and the expression of key genes during differentiation. Additionally, the “CytoTRACE2” package (version 1.1.0) (Yin et al., 2025) was used to predict the differentiation and developmental potential of the key cell type. Utilizing this model, CytoTRACE2 classified cells as either terminally differentiated (0) or totipotent (1). Finally, the communication among all cell types was explored by “cellchat” package (version 1.6.1) (Cao et al., 2024).

### 2.15 Statistical analysis

The R software (version 4.2.1) was adopted to conduct all analyses. A *P*-value less than 0.05 was deemed statistically significant.

## 3 Results

### 3.1 Descriptive characteristics

In this study, 22,086 ED-related ADE reports were obtained by FAERS database from Q1 2004 to Q1 2025. The demographic characteristics of these ADEs were displayed in Supplementary Table S3. As for age, reports had the most populations in 18–64 years group (n = 10,918, 49.4%). Most reports were obtained from consumer (n = 11,481, 52.0%), followed by the physician (n = 4,653, 21.1%). Furthermore, the most known outcomes were other serious outcomes (n = 7,868, 35.6%), while the second outcome was disability (n = 1,982, 9.0%). Otherwise, the United States was the main reported country for ED (n = 11,927, 57.16%), followed by the United Kingdom (n = 2,338, 11.20%) (Supplementary Figure S1A; Table 2). From 2004 to 2025, the peak of reports was 2015 (n = 1,753, 7.9%), and the second year was 2020 (n = 1,548, 7.0%) (Supplementary Figure S1B).

**TABLE 2:** Top 5 countries of patients with ADEs of ED.

Reported countries (top five)	N, %
United States	11,927 (57.16%)
United Kingdom	2,338 (11.20%)
Germany	1,112 (5.33%)
France	738 (3.54%)
Canada	578 (2.77%)

### 3.2 The selection of drug associated with ED

**FIGURE 2:**
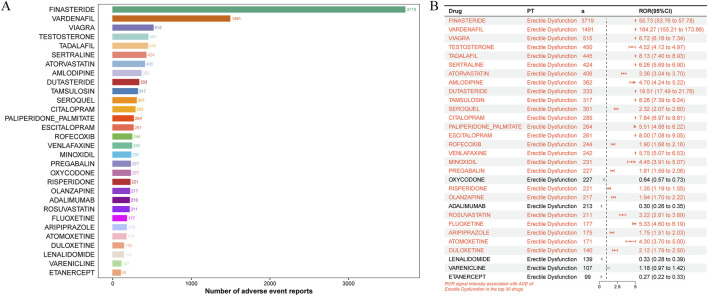
The drugs associated ED from FAERS database. **(A)** The forest plot for top 30 drugs associated ED. **(B)** Top 30 drugs with positive signals.

By computing the drugs that associated ED, we identified the top 30 drugs that were the suspects for causing ED (Figure 2A). Top 5 drugs within report numbers included finasteride (n = 3,719), vardenafil (n = 1,491), viagra (n = 515), testosterone (n = 450), and tadalafil (n = 445). Among these top 30 drugs, 25 had the positive signals, and top 5 drugs of signal intensity were vardenafil (ROR = 164.27, 95%CI = 155.21–173.86), finasteride (ROR = 55.73, 95%CI = 53.76–57.78), dutasteride (ROR = 19.51, 95%CI = 17.49–21.76), tamsulosin (ROR = 8.26, 95%CI = 7.39–9.24), and tadalafil (ROR = 8.13, 95%CI = 7.4–8.93). With the rising utilization of statin drug class (Omolaoye et al., 2022), we focused on the role of atorvastatin (ROR = 3.36, 95%CI = 3.04–3.7) and rosuvastatin (ROR = 3.22, 95%CI = 2.81–3.69) for ED (Figure 2B). To understand the physicochemical properties of these 2 drugs, corresponding ADMET was explored, and the results were displayed in Supplementary Table S4. From this table, we observed that these drugs had low gastrointestinal (GI) absorption and no blood-brain barrier (BBB) permeant, indicating that these drugs were difficult to pass through the BBB. Otherwise, they had the Pgp substrate, which meant they may be actively exported by Pgp. Furthermore, these 2 drugs had inhibitory action for common cytochrome P450 enzyme, such as CYP1A2 and CYP2C9. The log Kp value of atorvastatin and rosuvastatin was respectively −6.19 cm/s and −8.07 cm/s. Additionally, the toxicity of atorvastatin and rosuvastatin was also explored (Supplementary Table S5), and their predicted lethal dose (LD) was respectively 5,000 and 464 mg/kg, and the toxicity class was respectively 5 and 4 class, which indicated that these drugs were moderately toxic.

### 3.3 Identification and function prediction of shared targets between drugs and ED

**FIGURE 3:**
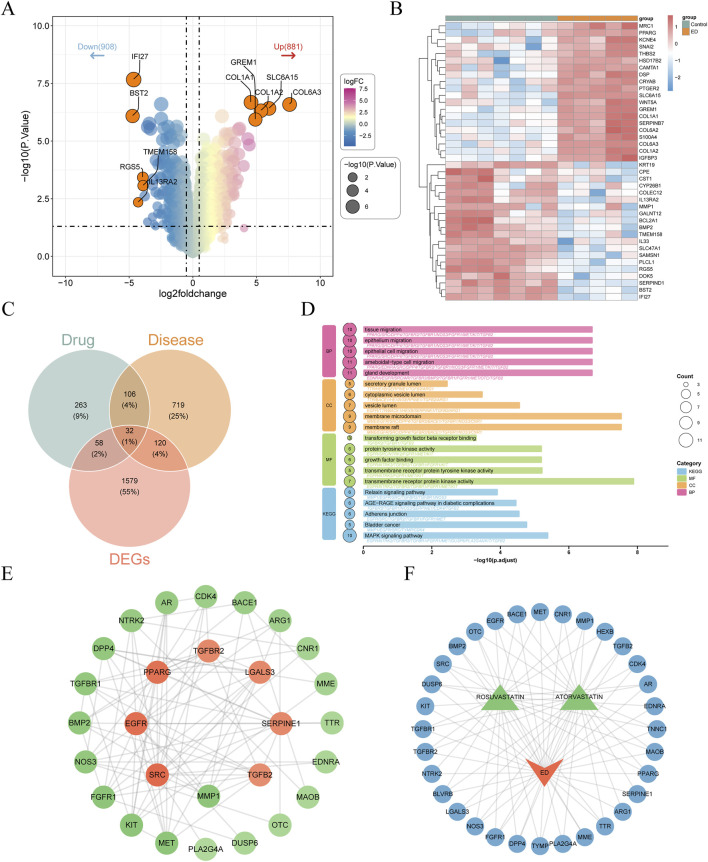
Identification and function prediction of shared targets between drugs and ED. **(A,B)** The volcano plot **(A)** and heat map **(B)** of differentially expressed genes (DEGs) between ED and control samples. **(C)** The intersection of drug targets, ED targets, and DEGs. **(D)** Gene Ontology (GO) and Kyoto Encyclopedia of Genes and Genomes (KEGG) enrichment analyses of intersection genes. **(E)** The protein-protein interaction (PPI) network of intersection genes. **(F)** A drug-ED-gene network. Orange represented ED, green denoted drugs, and blue was genes.

After summarizing and removing duplication, 459 drug-related targets and 977 ED-related targets were obtained from corresponding database. Differential expression analysis further identified 1,789 DEGs1, including 881 upregulated and 908 downregulated genes, between ED and control samples in the GSE10804 dataset (Figures 3A,B). The intersection of drug targets, ED targets, and DEGs1 yielded 32 intersection genes (Figure 3C). Enrichment analysis of intersection genes revealed 767 GO terms, such as gland development, membrane raft, and transmembrane receptor protein kinase activity, along with 47 KEGG pathways, containing MAPK signaling pathway, adherens junction, and relaxin signaling pathway (Figure 3D). PPI network of intersection genes was constructed with 28 nodes and 95 edges (Figure 3E). In this network, SRC had more correlations with other genes, such as EGFR, KIT, and PLA2G4A. The correlation of drugs, ED, and intersection genes was explored, and a network was created with 35 nodes and 84 edges (Figure 3F). Among these correlations, OTC, EGFR, BACE, MET, and other genes were associated with both drugs and ED.

### 3.4 FGFR1, SERPINE1, TGFB2, and TGFBR2 were identified as key genes in ED

**FIGURE 4:**
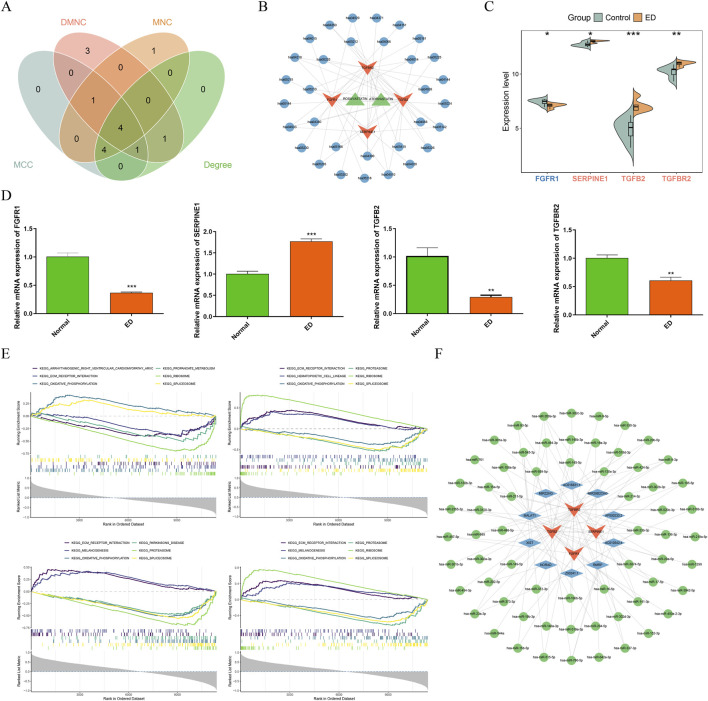
FGFR1, SERPINE1, TGFB2, and TGFBR2 were identified as key genes in ED. **(A)** The venn diagram of genes derived from 4 algorithms. **(B)** The drug-key gene-KEGG pathway network. Green denoted drugs, orange represented key genes, and blue denoted KEGG pthways. **(C,D)** The expression of key genes between ED and control samples in GSE10804 dataset and cell level. *P < 0.05, **P < 0.01, ***P < 0.001. **(E)** Gene set enrichment analysis of key genes. **(F)** The key gene-microRNA (miRNA)-long noncoding RNA (lncRNA) regulatory network. Orange was key genes, blue represented lncRNAs, and green denoted miRNAs.

Based on the results of PPI network, 4 algorithms-MCC, MNC, DMNC, and Degree-were utilized to select each top 10 genes (Supplementary Table S6). The intersection of these genes yielded 4 common genes: FGFR1, SERPINE1, TGFB2, and TGFBR2, which were identified as key genes (Figure 4A). According to above results, a drug-key gene-KEGG pathway network was constructed with 38 nodes and 61 edges (Figure 4B). In this network, atorvastatin and rosuvastatin targeted TGFBR2 and FGFR1, and these two targets were associated with cellular processes and environmental information processing pathways. Furthermore, the expression of key genes was explored, revealing that FGFR1 was significantly decreased, while SERPINE1 was markedly increased in both ED samples of GSE10804 dataset and cell model (Figures 4C,D). TGFB2 was significantly downregulated in the cell model (P < 0.01), which was inconsistent with the expression trend at the tissue level in bioinformatics analysis, and the core reason was the cell-specific regulation of TGFB2. To deeper understand the crucial roles of key genes in the development of ED, GSEA was conducted. The results illustrated that FGFR1, SERPINE1, TGFB2, and TGFBR2 respectively enriched 30, 28, 36, and 35 pathways. Common pathways enriched by all key genes included ECM receptor interaction, oxidative phosphorylation, and spliceosome (Figure 4E). These pathways work together to regulate disease development and progression. Additionally, the regulatory relationships of key genes were explored, and a key gene-miRNA-lncRNA regulatory network was developed with 78 nodes and 104 interaction pairs (Figure 4F). The associations in this network included FGFR1-hsa-miR-15a-5p-XIST, SERPINE1-hsa-miR-2355-3p-MALAT1, TGFB2-hsa-miR-141-3p-MIR29B2CHG, and TGFBR2-hsa-miR-106b-5p-MALAT1.

### 3.5 FGFR1-atorvastatin compound had stable structure

Furthermore, the molecular docking analysis was conducted between key genes and drugs, and the results showed that the binding energies were all less than −5 kcal/mol (Supplementary Table S7). Rosuvastatin correlated with the same residues-ASN-332, ARG-254, and LYS-381-of FGFR1 and TGFBR2. Atorvastatin was associated with the following residues: LEU-484, ASN-568, ARG-627, THR-658, THR-657, and ASN-659 of FGFR1; LYS-80, ARG-75, and ARG-115 of SERPINE1; LYS-277, THR-278, and GLY-276 of TGFB2; as well as LYS-291 and PHE-307 of TGFBR2 (Supplementary Figure S2A).

As shown in Supplementary Table S7, FGFR1 and atorvastatin had the lowest binding energy (−7.31 kcal/mol), so MD analysis was deployed across this compound. The results of RMSD, RMSF, and Rg all revealed the stable structure of the compound as the simulation proceeded (Supplementary Figures S2B–D). Additionally, the number of hydrogen across this compound was kept between 0 and 8 (Supplementary Figure S2E). The SASA fluctuated due to the combined conformational adjustment. However, the Buried SASA did not decrease to 0, indicating that the contact area of compound remained basically stable, and the binding between the two remained basically stable (Supplementary Figure S2F). Finally, their ΔE_MMPBSA_ value was −12.274 ± 2.326 kJ/mol (Supplementary Table S8), demonstrating higher binding energy and affinity. These findings all indicated that FGFR1 and atorvastatin had the excellent binding.

### 3.6 Fibroblasts were identified as key cell in ED

**FIGURE 5:**
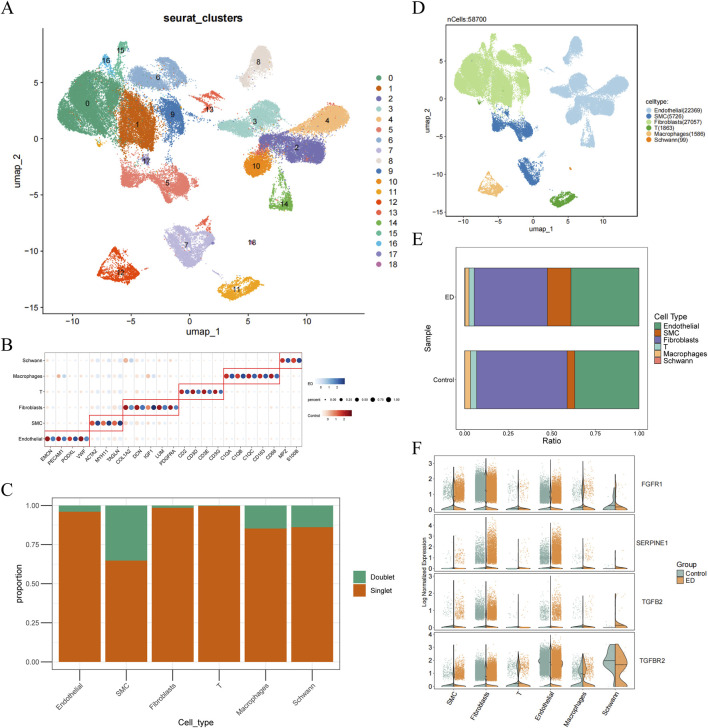
Fibroblasts were identified as key cell in ED. **(A)** Identification of cell clusters in ED. **(B)** Marker genes for cell clusters. **(C)** The proportion of singlet and doublets. **(D)** The annotation of cell types. **(E)** The proportion of cell types in ED and control samples. **(F)** The expression of key genes in cell types. ns, no significance, *P < 0.05, **P < 0.01, ***P < 0.001, ****P < 0.0001.

To investigate the single cell signature in ED samples, scRNA-seq analysis was conducted. After quality control, remaining 63,459 cells (38,430 ED cells and 25,029 control cells) for subsequent analysis (Supplementary Figure S3A). Among top 2,000 highly variable genes and top 30 PCs (Supplementary Figures S3B, C), 19 cell clusters were identified (Figure 5A). Based on marker genes, 6 cell types were annotated, and 58,700 cells were retained after removing 4,759 doublets (Figures 5B,C). These cell types included endothelial, Smooth Muscle Cell (SMC), fibroblasts, T cells, macrophages, and Schwann cells (Figure 5D). The proportion analysis showed that endothelial and fibroblasts had the high proportion in ED samples (Figure 5E). Further expression analysis revealed that all key genes had significant difference between ED and control samples in endothelial and fibroblasts cells (Figure 5F).

**FIGURE 6:**
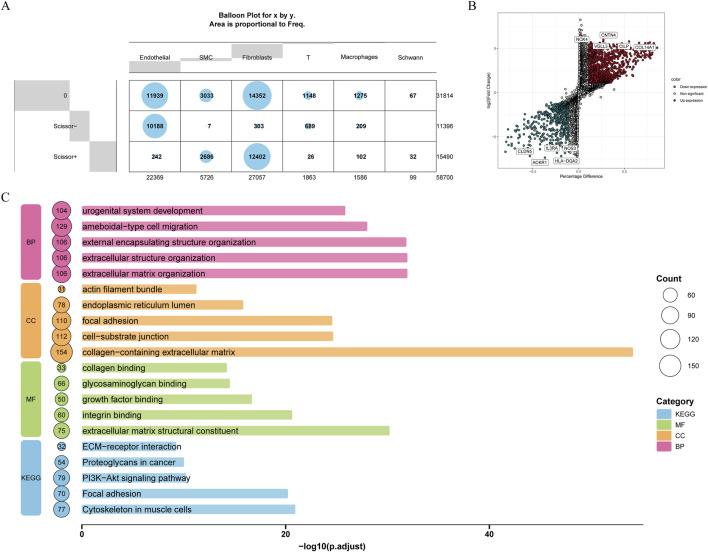
The correlation analysis between cell types with ED. **(A)** Correlation analysis of cell types with ED through Scissor package. **(B)** DEGs between Scissor+ and Scissor-cells. **(C)** GO and KEGG enrichment analysis of these DEGs.

Among these cell types, most endothelial and T cells were negatively correlated with ED, while majority fibroblasts and SMC cells were positively associated with ED (Figure 6A). Across Scissor+ and Scissor-cells, 1,814 DEGs2 were identified, comprising of 1,215 upregulated and 599 downregulated genes (Figure 6B). To explore the biological functions of DEGs2, enrichment analyses were conducted. The results revealed 1,845 GO terms, including extracellular matrix organization, collagen-containing extracellular matrix, and extracellular matrix structural constituent, as well as 108 KEGG pathways such as cytoskeleton in muscle cells, focal adhesion, and PI3K-Akt signaling pathway (Figure 6C). By combining the results of Scissor analysis and the expression of FGFR1, fibroblasts were identified as key cell for further analysis. The endothelial cell subpopulation was isolated from scRNA-seq data for independent analysis. A Wilcoxon test revealed that FGFR1 expression within endothelial cells of ED samples was markedly reduced compared to normal controls, a finding closely aligned with results from the GSE10804 bulk transcriptome dataset.

### 3.7 Fibroblasts had higher interactions with other cell types in ED

**FIGURE 7:**
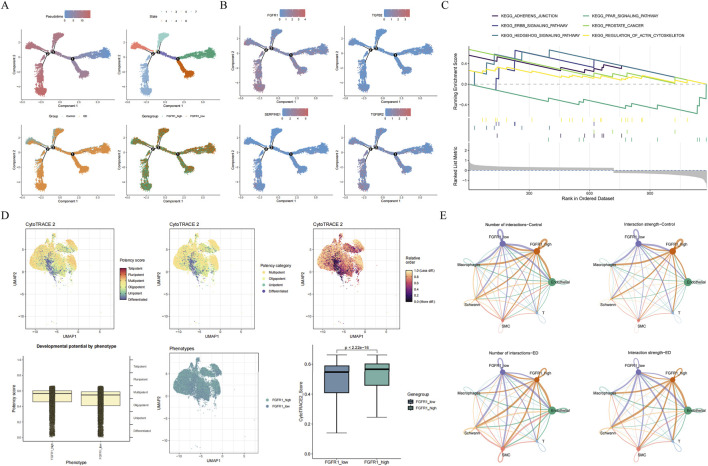
Pseudotime analysis and cell communication. **(A)** The differentiation station of fibroblasts. **(B)** The expression of key genes during differentiation. **(C)** GSEA for FGFR1 high and low expression groups. **(D)** Analysis of fibroblast totipotency. **(E)** Cell communication analysis across all cell types.

Pseudotime analysis of fibroblasts showed that 7 stages were existed during differentiation, and whole stages were found to be existed in ED samples (Figure 7A). During fibroblasts differentiation, FGFR1 and TGFBR2 were expressed in whole stage, while TGFB2 and SERPINE1 were expressed in the early and late stages (Figure 7B). Based on the expression of FGFR1, fibroblasts were divided into high and low expression groups. GSEA across these expression groups showed that ERBB signaling pathway, hedgehog signaling pathway, PPAR signaling pathway, and regulation of actin cytoskeleton were enriched (Figure 7C). These pathways were associated with cell proliferation, migration, and the progression of diseases. Additionally, fibroblasts were categorized into distinct differentiation stages, with the majority exhibiting potency scores close to 1 (Figure 7D). This indicated that the fibroblast populations predominantly resided in a pluripotent state of differentiation, representing incomplete differentiation. Concurrently, the potency score of the FGFR1 high-expression group surpassed that of the low-expression group, suggesting that cells in the high-expression group possessed greater totipotency. Cell communication analysis showed that fibroblast cells with high and low FGFR1 expression both had more and stronger interactions with other cells in ED samples (Figure 7E; Supplementary Figure S3D). These results indicated that fibroblast cells may play a crucial role in the development of ED through the correlated with other cells. Taken together, the dysregulation of intercellular communication of corpus cavernosum fibroblasts is the core cellular event of statin-associated ED, and its abnormal activation is closely mediated by the FGFR1/SERPINE1/TGFB2/TGFBR2 axis, which is the key molecular basis for fibroblast-driven local immunofibrotic disorder.

## 4 Discussion

ED is a prevalent clinical disorder impacting 30%–50% of men, with considerable negative implications on patients’ psychological wellbeing and quality of life (Xiong et al., 2024). The aetiology has significant variation, incorporating both common risk factors such as age, psychological influences, smoking, and alcohol use, as well as widely used drugs including diuretics, beta-blockers, antiandrogens, and psychiatric agents (Chen et al., 2019). In recent years, as statins have become more prominent in cardiovascular disease care, their potential reproductive harm has increasingly been revealed. In addition to the recognized link to heightened diabetes risk (Banihani, 2020), an expanding corpus of clinical and experimental studies suggests that statins may have multifaceted detrimental effects on male fertility: increasing sperm DNA fragmentation and abnormal sperm rates while diminishing sperm motility (Leite et al., 2018). Statin-induced sperm damage transpires via several mechanisms: (1) modification of sex hormones, especially testosterone; (2) triggering apoptosis of testicular germ cells; and (3) disrupting sperm lipid metabolism, hence affecting motility and morphology (Pons-Rejraji et al., 2014; Cai et al., 2008). Therefore, identifying statins linked to ED is essential for guaranteeing medication safety and alleviating this unpleasant effect. Clinical findings have associated atorvastatin use with the emergence or exacerbation of ED symptoms (Tian et al., 2025). Chen et al. conducted a meta-regression analysis that substantiated a causal association between genetically predicted atorvastatin use and ED, identifying atorvastatin as a risk factor for ED (Chen K. et al., 2024). Research involving animals demonstrates that 12 weeks of high-dose atorvastatin administration in mice led to a simultaneous decrease in the quantity of testicular supporting cells, spermatogonia, and spermatocytes (Akdeniz et al., 2021). Atorvastatin negatively impacts the reproductive system by diminishing levels of acid phosphatase, α-glucosidase, and L-carnitine in semen (Pons-Rejraji et al., 2014). Simultaneously, mice administered rosuvastatin demonstrated substantial decreases in both mature sperm count and daily sperm production (Leite et al., 2014). Despite the limited study on the association between rosuvastatin and ED, the recognized danger of atorvastatin in relation to ED warrants caution over the potential concerns of rosuvastatin. In conclusion, rigorous pharmacovigilance, molecular networks, and clinical cohort studies on atorvastatin and rosuvastatin offer potential for evidence-based guidance in risk stratification management in emergency departments.

In this context, we examined the target mechanisms of atorvastatin and rosuvastatin in ED, ultimately identified four key genes: FGFR1, SERPINE1, TGFB2, and TGFBR2. FGFR1 (fibroblast growth factor receptor 1) swiftly activates the PI3K-Akt-eNOS pathway in response to FGF stimulation of endothelial cells, hence preserving cavernous sinus endothelial homeostasis and nitric oxide bioavailability (Zhu et al., 2022). Recent investigations have demonstrated that FGFR1 regulates the course of testicular damage through the TAK1 signaling pathway (Zhang et al., 2025). In our analysis, the expression of FGFR1 showed significantly downregulated in ED samples. This downregulation results in interstitial cell atrophy, decreased germ cell numbers, and impaired differentiation (Li, 2021), potentially contributing to the development of ED. SERPINE1 (serine protease inhibitor clade E member 1), a principal inhibitor of the fibrinolytic system, has been implicated in neurotrauma-induced ED (Yin et al., 2021). Notably, our study observed an increased expression of this gene in ED. Elevated levels of SERPINE1 can may contribute to a hypercoagulable state and impair vascular endothelial function, disrupting the engorgement process of the corpus cavernosum and contributing to ED (Badran and Gozal, 2022). Moreover, SERPINE1 mRNA-binding protein 1 (SERBP1) has been established as a conserved regulator of stress granule clearance in somatic and male germ cells (Chen Z. et al., 2024). TGFB2 (transforming growth factor-beta 2), a prominent component of the TGF-β superfamily, meticulously modulates cell proliferation, differentiation, and apoptosis by paracrine or autocrine pathways, directly influencing the development of male reproductive organs (Young et al., 2015). Increased TGFB2 levels have been shown to correlate with the induction of death in male testicular gonadal cells and spermatogonia (Li X. et al., 2025), and to disrupt spermatogenesis by reversibly compromising the blood-testis barrier (Fan et al., 2012). The cell-specific regulation of TGFB2 is the main reason for the inconsistent expression trend between tissue level and cell model: the bioinformatics analysis is based on the bulk transcriptome data of the whole corpus cavernosum tissue, reflecting the overall expression characteristics of multiple cells such as fibroblasts, endothelial cells and immune cells; while the RT-qPCR is based on the single corpus cavernous endothelial cell model, and statin/palmitic acid-induced lipotoxic injury has an inhibitory effect on TGFB2 expression in endothelial cells, but a promoting effect on its expression in fibroblasts, which leads to the different expression trends of TGFB2 at different levels. TGFBR2 (TGFβ receptor 2), the high-affinity receptor for TGFβ2, mediates anti-fibrotic effects through miR-145 by suppressing TGFBR2, therefore reducing post-injury collagen deposition and cavernous smooth muscle cell death (Hong et al., 2025). Recent microarray analysis identifies TGFBR2 as a potential pathogenic gene in ED (Chen et al., 2025), affecting all stages of the disease process through its involvement in TGF-β-related pathways and signal transduction (Zhang Q. et al., 2023). In summary, these genes have a strong correlation with testis-related functional expression. Despite the limited current study on these genes in ED, their essential function in the testis demands immediate additional exploration of their relevance to ED.

Utilizing these key genes, we conducted GSEA to ascertain their significance in ED. The findings indicated that these key genes were concurrently abundant in the ECM receptor interaction, oxidative phosphorylation, and spliceosome pathways. It is well established that under normoxic conditions, cells convert chemical energy into the active chemical energy stored in ATP through oxidative phosphorylation via aerobic respiration, which serves as the primary energy source for sperm motility and spermatogonial stem cell self-renewal (Li et al., 2021; Liu et al., 2024). During hypoxia, the equilibrium between oxidative phosphorylation and glycolysis is altered, leading to diminished ATP synthesis. This hinders spermatogonial self-renewal and differentiation, results in meiotic failure, and precipitates a significant reduction in sperm motility (Chen et al., 2020; Zhou et al., 2018). Furthermore, ATP deficit impairs the contraction-relaxation cycle of smooth muscle, leading to phenotypic alteration and collagen accumulation, which fosters fibrosis and establishes a foundation for ED (Xu et al., 2020). The spliceosome, consisting of tiny nuclear ribonucleoproteins and other additional proteins, is crucial for the maturation of precursor messenger RNA in eukaryotic cells (Wilkinson et al., 2020). Studies demonstrate that splicing-associated proteins are abundantly expressed in spermatogonia, with alternative splicing playing a crucial role in the transition from mitosis to meiosis during mouse spermatogenesis (Liu et al., 2017). Recent investigations have emphasized the relationship between spliceosome component integrity and spermatogenesis. BUD31-mediated alternative splicing is crucial for preserving the primary spermatogonial stem cell reservoir and commencing spermatogenesis (Qin et al., 2023). Additionally, Wu H et al. discovered that spliceosome malfunction hinders spermatogonial differentiation, preventing germ cell maturation into spermatozoa (Wu et al., 2016). Moreover, the interaction of ECM receptors has been associated with the formation, differentiation, and maturation of male germ cells (Xi et al., 2024). While few studies have directly connected ECM receptor interaction or the spliceosome to ED, their established relationships with male germ cells highlight their importance in the male reproductive system. This necessitates additional inquiry into the influence of these pathways on ED.

In conclusion, our research utilizing the FAERS database substantiates that frequently given statins, atorvastatin and rosuvastatin, elevate the incidence of ED. Moreover, we have, for the first time, discovered key genes linked to these two drugs in ED, indicating that these genes may serve as viable targets for pharmacological intervention. Subsequent functional enrichment analysis indicated that the selected key genes were linked to pathways including ECM receptor interaction and spliceosome, so establishing the initial connection between these pathways and ED.

Abnormal regulation of ECM receptor interaction directly promotes the adhesion and proliferation of corpus cavernous fibroblasts and the deposition of extracellular matrix, leading to tissue fibrosis; oxidative phosphorylation disorder induces mitochondrial ATP synthesis deficiency, which impairs the contraction-relaxation function of corpus cavernous smooth muscle cells, and the above two are the key pathological links between these pathways and ED. The abnormal splicing function mediated by spliceosome disorder may affect the expression of key genes related to endothelial function and fibroblast activation, and indirectly participate in the occurrence of statin-associated ED. This offers a new viewpoint for clarifying the molecular pathways involved in statin-associated ED. As the central immunomodulatory cells of the corpus cavernosum, fibroblasts coordinate the local immune-fibrotic response in statin-associated ED through the FGFR1/SERPINE1/TGFB2/TGFBR2 axis. Abnormal activation of FGFR1 in fibroblasts upregulates the secretion of SERPINE1 and TGFB2, in which SERPINE1 promotes the recruitment and polarization of pro-inflammatory macrophages in the corpus cavernosum, leading to local inflammatory factor accumulation; TGFB2 binds to its receptor TGFBR2 to activate the downstream fibrotic signaling pathway, promoting collagen deposition and tissue fibrosis. In addition, the interaction between fibroblast-secreted factors and immune cells further forms a positive feedback loop of “inflammation-fibrosis”, which exacerbates the structural and functional damage of the corpus cavernosum and ultimately leads to ED. Nonetheless, our study possesses specific limitations. Initially, it is derived from the FAERS database, which depends on spontaneous reporting and is susceptible to underreporting, delayed reporting, and incomplete data. Furthermore, pharmacovigilance studies employing the FAERS database are fundamentally descriptive and cannot determine causality between these drugs and ED. The FAERS database only reveals a statistical association between atorvastatin/rosuvastatin and ED, and the causal relationship between them needs to be further verified by subsequent *in vivo* and *in vitro* intervention experiments. The FAERS data may have an inherent bias toward severe cases, as mild statin-associated ED cases are less likely to be reported, which may lead to an overestimation of the association intensity between statin use and ED. The palmitic acid-induced ED cell model only simulates the lipotoxic injury of corpus cavernous endothelial cells mediated by statins, and cannot fully replicate the comprehensive pathological changes of statin-associated ED *in vivo*. This study revealed four key genes in ED; nonetheless, their exact methods of action in this illness remain ambiguous. Therefore, additional *in vitro*, *in vivo*, and clinical investigations are necessary to elucidate the precise function of these genes in the etiology of ED.

## 5 Conclusion

This study initially investigated drugs linked to ED utilizing the FAERS database, specifically examining statins among these drugs and evaluating their toxicological profiles. Subsequently, key genes associated with ED were identified by network pharmacology and bioinformatics analyses of drugs and disorders. Molecular docking was conducted between these genes and the drugs, selecting complexes with the lowest binding energies for MD analysis to assess structure stability. Simultaneously, GSEA and regulatory relationship prediction were conducted on key genes to clarify their functional pathways. Ultimately, scRNA-seq of ED was performed to delineate cellular characteristics and identify essential cell types. These techniques aim to offer innovative therapeutic targets and conceptual frameworks for the management of ED.

## Data Availability

Publicly available datasets were analyzed in this study. This data can be found here: The data for this study were sourced from FAERS database (https://www.fda.gov/drugs) and GEO database (GSE10804, GSE206528, https://www.ncbi.nlm.nih.gov/gds/).

## Supplementary material

SUPPLEMENTARY FIGURE S1 Signals detection for ED. **(A)** Country distribution of reports for ED. **(B)** Distribution of reports for ED from 2004 to 2025.

SUPPLEMENTARY FIGURE S2 FGFR1-atorvastatin compound had stable structure. **(A)** Molecular docking between key genes with corresponding drugs. **(B-D)** MoThe results of root mean square deviation (RMSD) **(B)**, root mean square fluctuation (RMSF) **(C)**, and radius of gyration (Rg) **(D)** for FGFR1-atorvastatin compound. **(E)** The number of Hbone. **(F)** The Buried solvent accessible surface area (SASA) result.

SUPPLEMENTARY FIGURE S3 Single-cell RNA sequencing (scRNA-seq) analysis in ED. **(A)** The quality control for sc-RNA seq data. **(B)** Top 2,000 highly variable genes. **(C)** Top 30 principle components. **(D)** The interaction of each cell type with others.
