# Supplementary material for: Dysregulated fibroblast–immune crosstalk drives statin-associated erectile dysfunction: integrative evidence from pharmacovigilance and single-cell transcriptomics
Source: Front Pharmacol. 2026 Mar 24;17:1787039. doi: 10.3389/fphar.2026.1787039 (PMC13161919; doi:10.3389/fphar.2026.1787039)
Supplement: Supplementary file 1 [file Table1.docx]

**Supplementary Table 1.** Four-grid table for signal detection

|  | Target adverse drug event | Other adverse drug event | Sums |
| --- | --- | --- | --- |
| Drugs | a | b | a+b |
| Other drugs | c | d | c+d |
| Sums | a+c | b+d | a+b+c+d |

**Supplementary Table 2.** Overview of the main algorithms used for signal detection

| Algorithms | Equation | Criteria |
| --- | --- | --- |
| ROR | ROR=ad/bc | 95% CI (lower limit) > 1, N ≥ 3 |
|  | 95%CI=eln(ROR)±1.96(1/a+1/b+1/c+1/d)^0.5 |  |
| PRR | PRR=a(c+d)/c/(a+b) | N ≥ 3,PRR ≥ 2, χ2 ≥ 4 |
|  | 95%CI=eln(PRR)±1.96(1/a+1/(a+b)+1/c+1/(c+d))^0.5 |  |
|  | χ2=[(ad-bc)^2](a+b+c+d)/[(a+b)(c+d)(a+c)(b+d)] |  |
| BCPNN | IC=log2a(a+b+c+d)/(a+c)/(a+b) | IC025 > 0 |
|  | 95%CI=E(IC) ± 2V(IC)^0.5 |  |
|  | r=(a+b+c+d)^2/(a+b+1)/(a+c+1) |  |
|  | E(IC)=log2a(a+b+c+d)^2/(a+b+c+d+r)/(a+b)/(a+c) |  |
|  | V(IC)=1/ln2{(b+c+d+r-1)/(a+1)/(a+b+c+d+r+1)+(2+b+c+2d)/(a++b+1)/(a+b+c+d+r+3)} |  |
|  | IC025=E(IC)- 2V(IC)^0.5 |  |
| MGPS | EBGM=a(a+b+c+d)/(a+c)/(a+b) | EBGM05 > 2 |
|  | EBGM05=eln(EBGM)-1.96(1/a+1/b+1/c+1/d)^0.5 |  |

ROR: Reporting odds ratio; PRR: Proportional reporting ratio; BCPNN: Bayesian confidence propagation neural network; MGPS: Multi-item gamma Poisson shrinker; EBGM: Empirical

Bayesian ensemble mean; IC: Information component; CI: Confidence interval.

**Supplementary Table 3.** Clinical characteristics of patients with adverse drug events (ADEs) of erectile dysfunction (ED)

| Characteristics | Variable | Number of reports | percentage |
| --- | --- | --- | --- |
| Gender | Male | 22,086 | 100% |
| Weight | < 50 kg | 33 | 0.1% |
|  | > 100 kg | 1,779 | 8.1% |
|  | 50~100 kg | 7,238 | 32.8% |
|  | Unknown | 13,036 | 59.0% |
| Age | < 18 | 260 | 1.2% |
|  | > 85 | 56 | 0.3% |
|  | 18 ~ 64 | 10,918 | 49.4% |
|  | 65 ~ 85 | 2,850 | 12.9% |
|  | Unknown | 8,002 | 36.2% |
| Reporter’s occupation | Consumer (CN) | 11,481 | 52.0% |
|  | Health-professional (HP) | 1,053 | 4.8% |
|  | Lawyer (LW) | 514 | 2.3% |
|  | Physician (MD) | 4,653 | 21.1% |
|  | Other health-professional (OT) | 1,694 | 7.7% |
|  | Pharmacist (PH) | 666 | 3.0% |
|  | Unknown | 2,023 | 9.2% |
| Reported time | 2004 | 795 | 3.6% |
|  | 2005 | 666 | 3.0% |
|  | 2006 | 779 | 3.5% |
|  | 2007 | 381 | 1.7% |
|  | 2008 | 600 | 2.7% |
|  | 2009 | 562 | 2.5% |
|  | 2010 | 931 | 4.2% |
|  | 2011 | 800 | 3.6% |
|  | 2012 | 958 | 4.3% |
|  | 2013 | 851 | 3.9% |
|  | 2014 | 1,185 | 5.4% |
|  | 2015 | 1,753 | 7.9% |
|  | 2016 | 1,143 | 5.2% |
|  | 2017 | 955 | 4.3% |
|  | 2018 | 855 | 3.9% |
|  | 2019 | 1,095 | 5.0% |
|  | 2020 | 1,548 | 7.0% |
|  | 2021 | 1,492 | 6.8% |
|  | 2022 | 1,376 | 6.2% |
|  | 2023 | 1,439 | 6.5% |
|  | 2024 | 1,442 | 6.5% |
|  | 2025 | 480 | 2.2% |
| Outcome | Death (DE) | 222 | 1.0% |
|  | Disability (DS) | 1,982 | 9.0% |
|  | Hospitalization (HO) | 1,397 | 6.3% |
|  | Life -threaten  ing (LT) | 376 | 1.7% |
|  | Other serious outcomes (OT) | 7,868 | 35.6% |
|  | Required intervention (RI) | 22 | 0.1% |
|  | Missing | 10,211 | 46.2% |

**Supplementary Table 4.** The ADMET of atorvastatin and rosuvastatin

|  | Atorvastatin | Rosuvastatin |
| --- | --- | --- |
| GI absorption | Low | Low |
| BBB permeant | No | No |
| Pgp substrate | Yes | Yes |
| CYP1A2 inhibitor | No | No |
| CYP2C19 inhibitor | Yes | No |
| CYP2C9 inhibitor | No | No |
| CYP2D6 inhibitor | Yes | No |
| CYP3A4 inhibitor | Yes | No |
| log Kp (cm/s) | -6.19 | -8.07 |

**Supplementary Table 5.** The toxicity analysis of atorvastatin and rosuvastatin

| Name | Atorvastatin | Rosuvastatin |
| --- | --- | --- |
| Predicted LD50(mg/kg) | 5000 | 464 |
| Predicted Toxicity Class | 5 | 4 |
| Prediction accuracy | 67.38% | 54.26% |
| Molweight | 558.64 | 481.51 |
| Number of hydrogen bond acceptors | 6 | 9 |
| Number of hydrogen bond donors | 4 | 3 |
| Number of atoms | 41 | 33 |
| Number of bonds | 44 | 34 |
| Number of rotable bonds | 13 | 10 |
| Molecular refractivity | 158.26 | 123.4 |
| Topological Polar Surface Area | 111.79 | 149.3 |
| octanol/water partition coefficient(logP) | 6.39 | 3.48 |

**Supplementary Table 6.** The top 10 genes for each algorithm

| **MCC** | **DMNC** | **MNC** | **Degree** |
| --- | --- | --- | --- |
| SRC | TGFB2 | SRC | SRC |
| PPARG | BMP2 | PPARG | PPARG |
| TGFB2 | MMP1 | TGFB2 | TGFB2 |
| KIT | KIT | FGFR1 | MMP1 |
| FGFR1 | FGFR1 | SERPINE1 | KIT |
| SERPINE1 | AR | MET | FGFR1 |
| TGFBR1 | SERPINE1 | TGFBR1 | SERPINE1 |
| LGALS3 | CDK4 | LGALS3 | LGALS3 |
| EGFR | TGFBR1 | EGFR | EGFR |
| TGFBR2 | TGFBR2 | TGFBR2 | TGFBR2 |

MCC: Maximal Clique Centrality; MNC: Maximum Neighborhood Component; DMNC: Density of Maximum Neighborhood Component.

**Supplementary Table 7.** The molucular docking of key genes with drugs

| Genes | UniProt | PDB | Drugs | Drug CID | Binding energy  (kcal/mol) | Number of hydrogen |
| --- | --- | --- | --- | --- | --- | --- |
| FGFR1 | P11362 | 5EW8 | Rosuvastatin | CID446157 | -5.42 | 5 |
| TGFBR2 | P37173 | 8YGZ | Rosuvastatin | CID446157 | -6.01 | 4 |
| FGFR1 | P11362 | 5EW8 | Atorvastatin | CID60823 | -7.31 | 7 |
| SERPINE1 | P05121 | 1DB2 | Atorvastatin | CID60823 | -5.34 | 3 |
| TGFBR2 | P37173 | 8YGZ | Atorvastatin | CID60823 | -5.75 | 2 |
| TGFB2 | P61812 | 8FXV | Atorvastatin | CID60823 | -6.3 | 6 |

**Supplementary Table 8.** The binding energy and constitution of FGFR1-atorvastatin

| Complex | Protein-Ligand |
| --- | --- |
| ΔE_vdw_ | -149.223±2.985 |
| ΔE_ele_ | -110.041±1.414 |
| ΔE_pol_ | 273.657±6.962 |
| ΔE_nonpol_ | -26.667±0.59 |
| ΔE_MMPBSA_ | -12.274±2.326 |
| -TΔS | 27.226±3.462 |
| ΔG_bind_(ΔEvdw+ΔEele+ΔEpol+ΔEnonpol-TΔS) | 14.953±3.082 |
