# Supplementary figures and images for: Dysregulated fibroblast–immune crosstalk drives statin-associated erectile dysfunction: integrative evidence from pharmacovigilance and single-cell transcriptomics
Source: Front Pharmacol. 2026 Mar 24;17:1787039. doi: 10.3389/fphar.2026.1787039 (PMC13161919; doi:10.3389/fphar.2026.1787039)

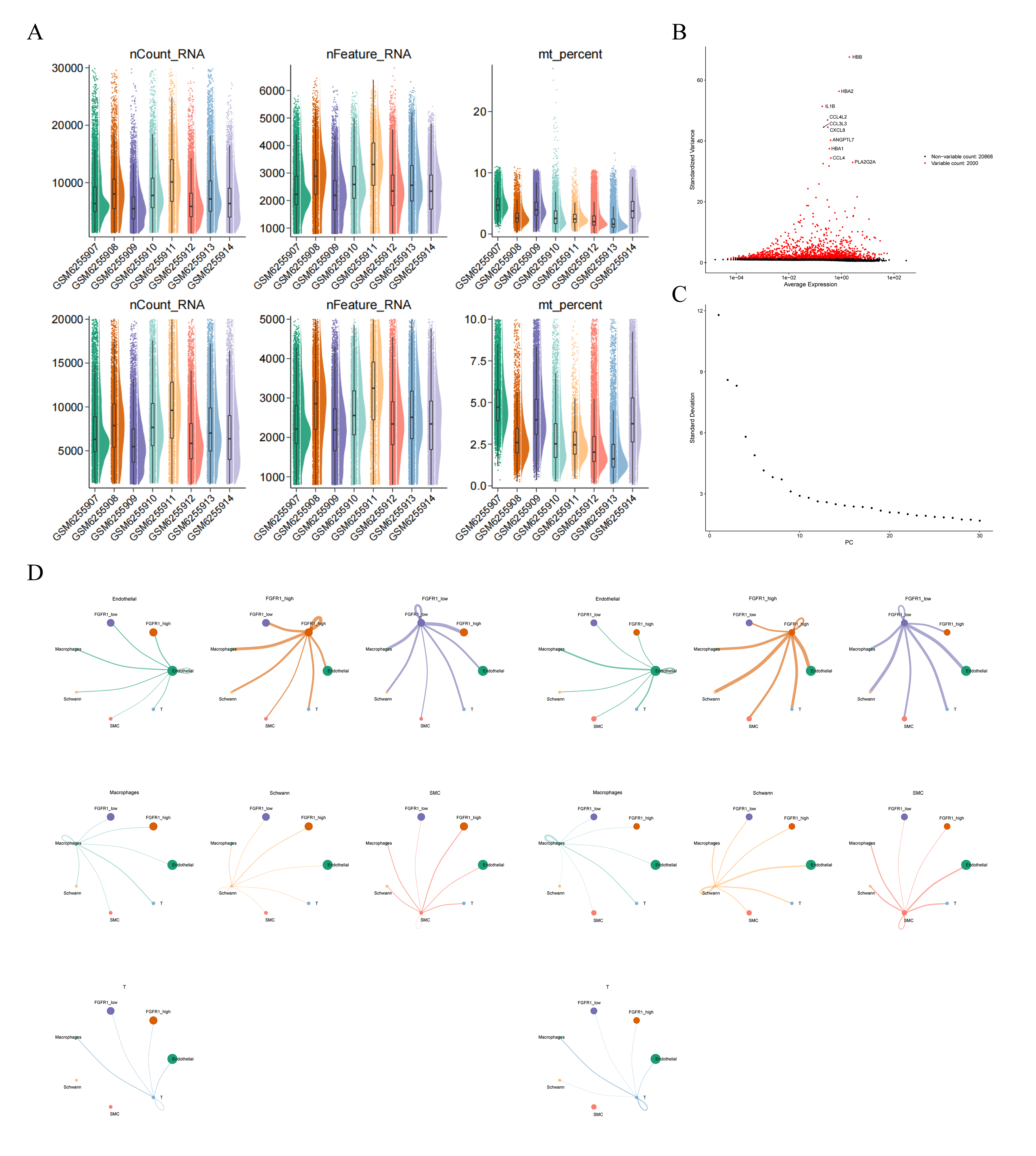

Supplement: Supplementary file 2 [file Image3.tif]

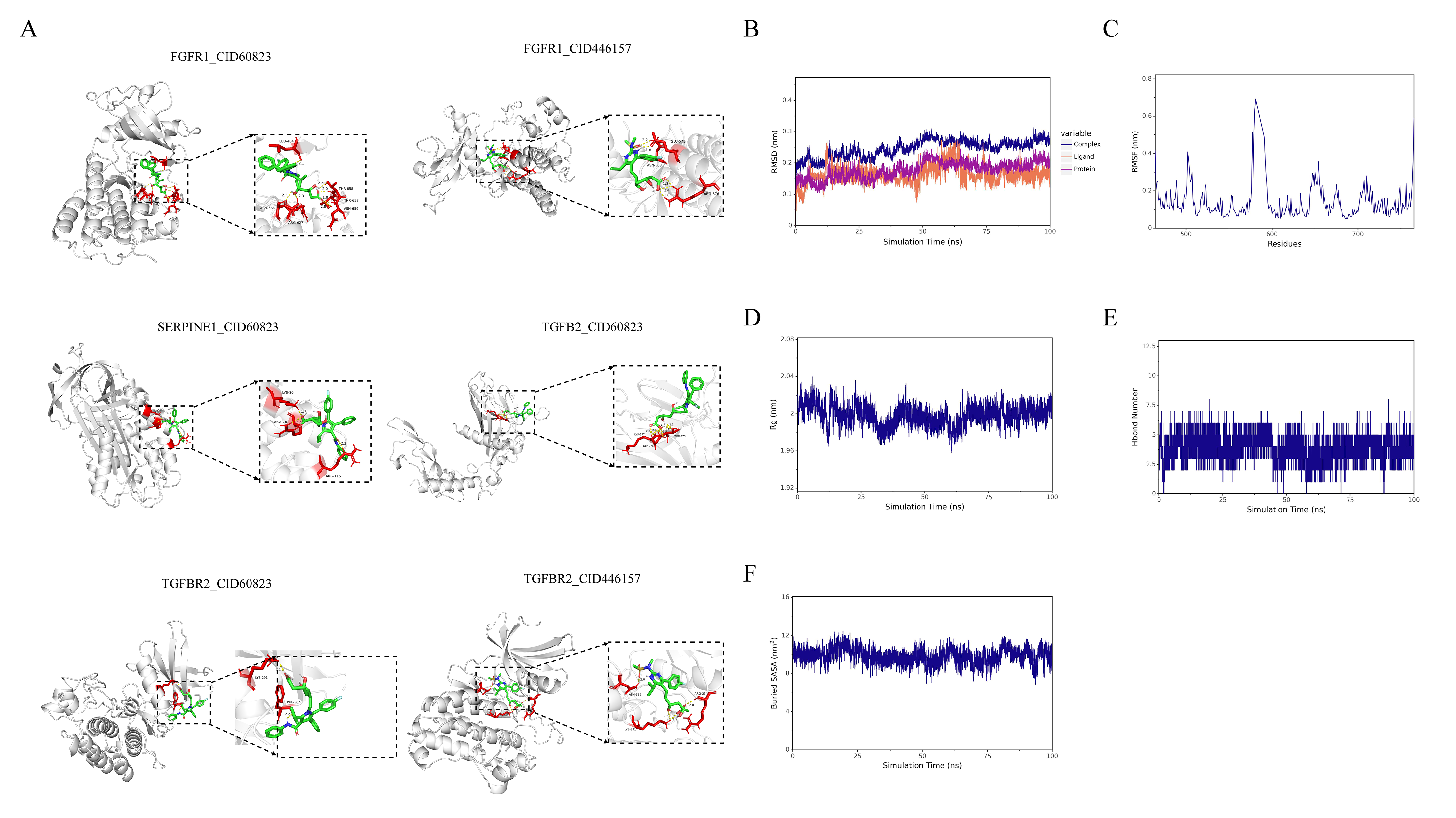

Supplement: Supplementary file 3 [file Image2.tif]

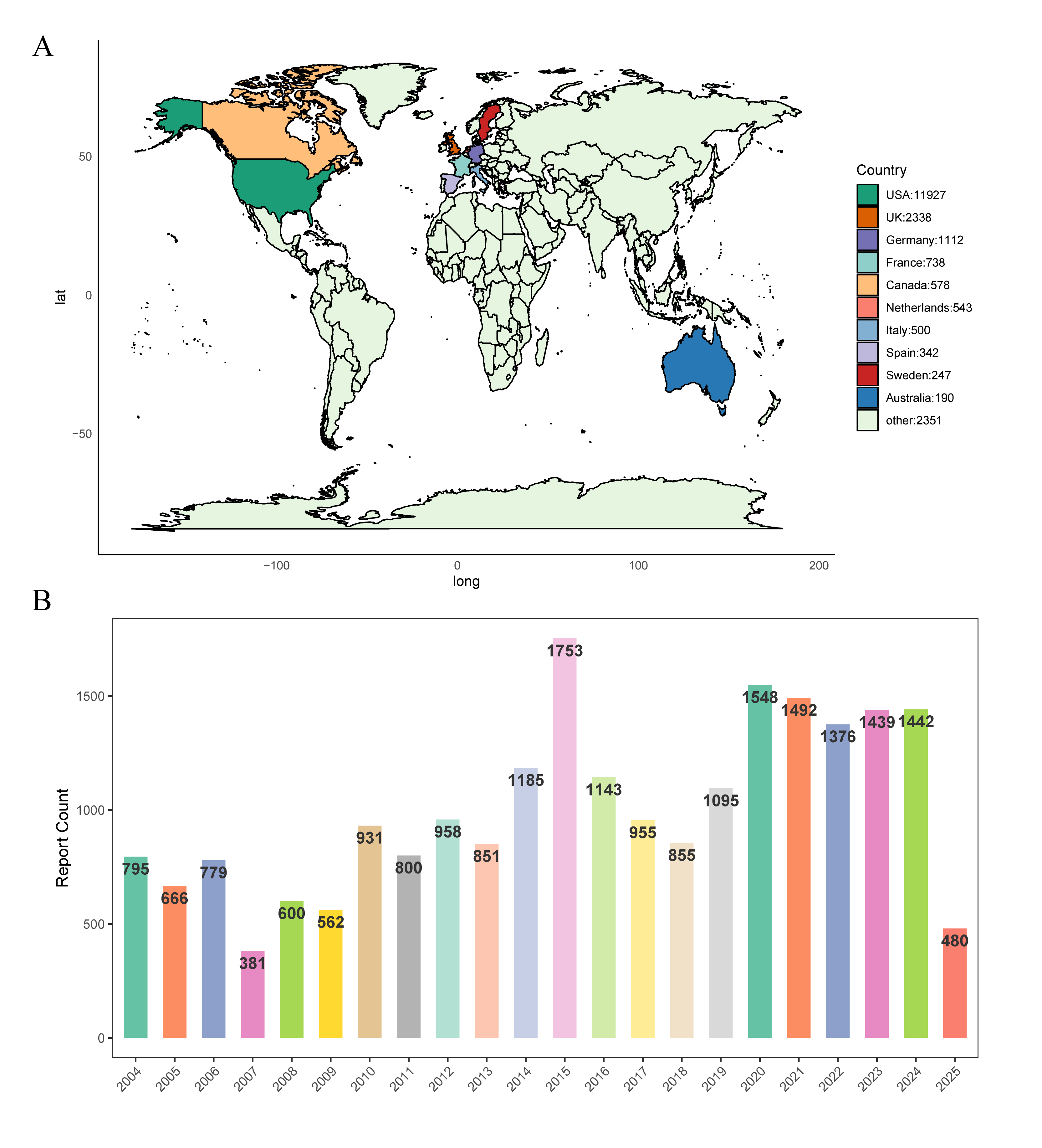

Supplement: Supplementary file 4 [file Image1.tif]
